# Supplementary material for: Forelimb musculoskeletal-tendinous growth in frogs
Source: PeerJ. 2020 Feb 25;8:e8618. doi: 10.7717/peerj.8618 (PMC7047859; doi:10.7717/peerj.8618)
Supplement: Table S2 [file peerj-08-8618-s002.docx]

| Variable | Expected allometry coefficient | Observed allometry coefficient | Observed departure | Untrimmed | | | | Trimmed | | | |
| --- | --- | --- | --- | --- | --- | --- | --- | --- | --- | --- | --- |
|  |  |  |  | Resampled allometry coefficient | Bias | 95 % CI | Growth trend | Resampled allometry coefficient | Bias | 95 % CI | Growth trend |
| LT | 0.21 | 0.815 | 0.605 | 0.227 | -0.003 | 0.215-0.238 | + | 0.226 | -0.002 | 0.218-0.234 | + |
| HL | 0.21 | 0.192 | -0.018 | 0.185 | -0.002 | 0.143-0.225 | = | 0.182 | -0.001 | 0.147-0.217 | = |
| RUL | 0.21 | 0.173 | -0.037 | 0.236 | -0.002 | 0.211-0.260 | = | 0.237 | -0.003 | 0.222-0.252 | + |
| SM | 0.21 | 0.172 | -0.038 | 0.248 | -0.002 | 0.211-0.283 | = | 0.251 | -0.003 | 0.223-0.277 | + |
| SMTL | 0.21 | 0.054 | -0.159 | 0.283 | -0.005 | 0.180-0.385 | = | 0.291 | -0.009 | 0.219-0.362 | + |
| Hlat | 0.21 | 0.171 | -0.042 | 0.254 | -0.003 | 0.235-0.272 | + | 0.251 | -0.001 | 0.236-0.264 | + |
| HlatTL | 0.21 | 0.025 | -0.188 | 0.116 | -0.001 | 0.010-0.222 | = | 0.134 | -0.01 | 0.059-0.209 | - |
| Hmed | 0.21 | 0.16 | -0.053 | 0.274 | -0.003 | 0.215-0.333 | + | 0.261 | 0.004 | 0.214-0.307 | + |
| HmedTL | 0.21 | 0.033 | -0.18 | 0.178 | -0.007 | 0.025-0.331 | = | 0.092 | 0.036 | 0.007-0.177 | - |
| Edig | 0.21 | 0.189 | -0.024 | 0.255 | -0.003 | 0.235-0.275 | + | 0.254 | -0.002 | 0.236-0.270 | + |
| Ecul | 0.21 | 0.187 | -0.026 | 0.29 | -0.003 | 0.259-0.322 | + | 0.295 | -0.006 | 0.274-0.317 | + |
| EculT | 0.21 | 0.018 | -0.195 | 0.198 | -0.003 | 0.115-0.281 | = | 0.198 | -0.003 | 0.144-0.252 | = |
| Ecr | 0.21 | 0.108 | -0.106 | 0.209 | -0.001 | 0.139-0.279 | = | 0.217 | -0.005 | 0.168-0.264 | = |
| EcrT | 0.21 | 0.035 | -0.179 | 0.276 | -0.004 | 0.188-0.363 | = | 0.315 | -0.023 | 0.251-0.379 | + |
| C | 0.21 | 0.081 | -0.133 | 0.185 | -0.002 | 0.148-0.221 | = | 0.174 | 0.003 | 0.148-0.200 | - |
| CTL | 0.21 | 0.153 | -0.06 | 0.212 | -0.002 | 0.190-0.234 | = | 0.217 | -0.004 | 0.200-0.233 | = |
| Fdc | 0.21 | 0.167 | -0.046 | 0.269 | -0.002 | 0.246-0.291 | + | 0.272 | -0.004 | 0.253-0.291 | + |
| FdcT | 0.21 | 0.003 | -0.21 | 0.028 | -0.001 | -0.072-.128 | - | 0.021 | 0.003 | -0.058-0.101 | - |
| Fcul | 0.21 | 0.133 | -0.08 | 0.238 | -0.002 | 0.197-0.278 | = | 0.245 | -0.006 | 0.219-0.270 | + |
| FculT | 0.21 | 0.004 | -0.209 | -0.068 | 0.044 | -0.165-.030 | - | -0.068 | 0.044 | -0.144-0.008 | - |
| Fcr | 0.21 | 0.143 | -0.07 | 0.245 | -0.003 | 0.218-0.271 | + | 0.235 | 0.002 | 0.218-0.251 | + |
| FcrT | 0.21 | 0.007 | -0.206 | 0.071 | -0.000 | 0.003-0.139 | - | 0.061 | 0.004 | 0.008-0.114 | - |
