# Supplementary material for: Forelimb musculoskeletal-tendinous growth in frogs
Source: PeerJ. 2020 Feb 25;8:e8618. doi: 10.7717/peerj.8618 (PMC7047859; doi:10.7717/peerj.8618)
Supplement: Table S3 [file peerj-08-8618-s003.docx]

| Variables | Expected allometry coefficient | Observed allometry coefficient | Observed departure | Untrimmed | | | | Trimmed | | | |
| --- | --- | --- | --- | --- | --- | --- | --- | --- | --- | --- | --- |
|  |  |  |  | Resampled allometry coefficient | Bias | 95 % CI | Growth trend | Resampled allometry coefficient | Bias | 95 % CI | Growth trend |
| LT | 0.21 | 0.754 | 0.541 | 0.192 | -0.012 | 0.105-0.279 | = | 0.215 | -0.024 | 0.174-0.256 | = |
| HL | 0.21 | 0.249 | 0.036 | 0.252 | -0.020 | 0.126-0.377 | = | 0.263 | -0.026 | 0.214-0.311 | + |
| RUL | 0.21 | 0.164 | -0.049 | 0.242 | -0.021 | 0.076-0.408 | = | 0.254 | -0.027 | 0.204-0.303 | = |
| SM | 0.21 | 0.172 | -0.042 | 0.245 | -0.019 | 0.139-0.35 | = | 0.244 | -0.019 | 0.201-0.286 | = |
| SMTL | 0.21 | 0.028 | -0.186 | 0.142 | -0.017 | 0.054-0.230 | = | 0.108 | 0.000 | 0.061-0.155 | - |
| Hlat | 0.21 | 0.170 | -0.043 | 0.246 | -0.017 | 0.144-0.348 | = | 0.267 | -0.027 | 0.224-0.309 | + |
| HlatTL | 0.21 | 0.023 | -0.190 | 0.046 | 0.007 | -0.025-0.118 | - | 0.082 | -0.010 | 0.048-0.115 | - |
| Hmed | 0.21 | 0.073 | -0.141 | 0.174 | -0.020 | -0.025-0.374 | = | 0.182 | -0.024 | 0.118-0.245 | = |
| HmedTL | 0.21 | -0.366 | -0.579 | 0.614 | -0.058 | 0.051-1.177 | = | 0.250 | 0.123 | 0.053-0.447 | = |
| Edig | 0.21 | 0.157 | -0.056 | 0.247 | -0.021 | 0.119-0.375 | = | 0.243 | -0.019 | 0.205-0.280 | = |
| Ecul | 0.21 | 0.161 | -0.052 | 0.282 | -0.012 | 0.174-0.389 | = | 0.320 | -0.031 | 0.240-0.399 | + |
| EculT | 0.21 | 0.014 | -0.199 | 0.115 | 0.008 | -0.021-0.252 | = | 0.180 | -0.024 | 0.120-0.240 | = |
| Ecr | 0.21 | 0.104 | -0.110 | 0.180 | -0.010 | 0.057-0.302 | = | 0.228 | -0.034 | 0.149-0.306 | = |
| EcrT | 0.21 | 0.039 | -0.174 | 0.245 | 0.013 | -0.031-0.519 | = | 0.308 | -0.018 | 0.266-0.349 | + |
| C | 0.21 | 0.053 | -0.161 | 0.173 | -0.026 | -0.002-0.349 | = | 0.135 | -0.006 | 0.109-0.161 | - |
| CTL | 0.21 | 0.174 | -0.040 | 0.236 | -0.017 | 0.142-0.329 | = | 0.243 | -0.021 | 0.205-0.281 | = |
| Fdc | 0.21 | 0.125 | -0.088 | 0.246 | -0.024 | 0.072-0.420 | = | 0.251 | -0.026 | 0.212-0.289 | = |
| FdcT | 0.21 | 0.020 | -0.193 | 0.205 | -0.006 | 0.136-0.273 | = | 0.192 | 0.000 | 0.141-0.243 | = |
| Fcul | 0.21 | 0.123 | -0.090 | 0.272 | -0.021 | 0.151-0.393 | = | 0.282 | -0.027 | 0.239-0.326 | + |
| FculT | 0.21 | 0.027 | -0.186 | 0.162 | 0.021 | -0.106-0.431 | = | 0.240 | -0.019 | 0.112-0.369 | = |
| Fcr | 0.21 | 0.124 | -0.089 | 0.284 | -0.033 | 0.094-0.473 | = | 0.242 | -0.012 | 0.224-0.26 | + |
| FcrT | 0.21 | 0.017 | -0.196 | 0.138 | 0.004 | 0.001-0.276 | = | 0.168 | -0.011 | 0.074-0.261 | = |
