# Supplementary material for: Forelimb musculoskeletal-tendinous growth in frogs
Source: PeerJ. 2020 Feb 25;8:e8618. doi: 10.7717/peerj.8618 (PMC7047859; doi:10.7717/peerj.8618)
Supplement: Table S4 [file peerj-08-8618-s004.docx]

| Variables | Expected allometry coefficient | Observed allometry coefficient | Observed departure | Untrimmed | | | | Trimmed | | | |
| --- | --- | --- | --- | --- | --- | --- | --- | --- | --- | --- | --- |
|  |  |  |  | Resampled allometry coefficient | Bias | 95 % CI | Growth trend | Resampled allometry coefficient | Bias | 95 % CI | Growth trend |
| LT | 0.21 | 0.842 | 0.629 | 0.215 | -0.001 | 0.199-0.231 | = | 0.216 | -0.002 | 0.206-0.225 | = |
| HL | 0.21 | 0.215 | 0.002 | 0.229 | -0.002 | 0.187-0.272 | = | 0.233 | -0.004 | 0.222-0.244 | + |
| RUL | 0.21 | 0.148 | -0.065 | 0.214 | -0.001 | 0.199-0.229 | = | 0.215 | -0.001 | 0.203-0.226 | = |
| SM | 0.21 | 0.163 | -0.050 | 0.281 | -0.003 | 0.239-0.322 | + | 0.273 | 0.001 | 0.252-0.293 | + |
| SMTL | 0.21 | 0.046 | -0.167 | 0.226 | -0.002 | 0.193-0.258 | = | 0.228 | -0.003 | 0.205-0.249 | = |
| Hlat | 0.21 | 0.161 | -0.052 | 0.253 | -0.001 | 0.193-0.313 | = | 0.255 | -0.002 | 0.247-0.263 | + |
| HlatTL | 0.21 | 0.036 | -0.177 | 0.168 | -0.001 | 0.076-0.259 | = | 0.192 | -0.013 | 0.162-0.223 | = |
| Hmed | 0.21 | 0.094 | -0.119 | 0.213 | -0.003 | 0.171-0.256 | = | 0.206 | 0.001 | 0.181-0.229 | = |
| HmedTL | 0.21 | 0.014 | -0.199 | 0.122 | -0.001 | 0.094-0.149 | - | 0.107 | 0.007 | 0.094-0.120 | - |
| Edig | 0.21 | 0.182 | -0.031 | 0.269 | -0.002 | 0.251-0.287 | + | 0.266 | -0.001 | 0.255-0.276 | + |
| Ecul | 0.21 | 0.129 | -0.085 | 0.229 | -0.001 | 0.196-0.262 | = | 0.235 | -0.003 | 0.221-0.248 | + |
| EculT | 0.21 | 0.018 | -0.195 | 0.100 | 0.002 | 0.010-0.190 | - | 0.080 | 0.012 | 0.028-0.131 | - |
| Ecr | 0.21 | 0.112 | -0.101 | 0.266 | -0.002 | 0.226-0.306 | + | 0.259 | 0.002 | 0.239-0.279 | + |
| EcrT | 0.21 | 0.028 | -0.185 | 0.197 | -0.002 | 0.069-0.324 | = | 0.121 | 0.036 | 0.083-0.158 | - |
| C | 0.21 | 0.118 | -0.095 | 0.217 | 0.000 | 0.193-0.240 | = | 0.225 | -0.004 | 0.210-0.240 | = |
| CTL | 0.21 | 0.168 | -0.045 | 0.223 | -0.002 | 0.194-0.252 | = | 0.227 | -0.004 | 0.217-0.238 | + |
| Fdc | 0.21 | 0.136 | -0.077 | 0.235 | -0.001 | 0.202-0.267 | = | 0.237 | -0.002 | 0.219-0.254 | + |
| FdcT | 0.21 | 0.023 | -0.190 | 0.177 | -0.003 | 0.12-0.233 | = | 0.157 | 0.006 | 0.126-0.188 | - |
| Fcul | 0.21 | 0.133 | -0.080 | 0.220 | 0.000 | 0.176-0.263 | = | 0.229 | -0.005 | 0.196-0.262 | = |
| FculT | 0.21 | 0.032 | -0.181 | 0.180 | -0.002 | 0.122-0.238 | = | 0.167 | 0.004 | 0.130-0.204 | - |
| Fcr | 0.21 | 0.117 | -0.096 | 0.246 | -0.001 | 0.197-0.293 | = | 0.225 | 0.009 | 0.207-0.243 | = |
| FcrT | 0.21 | 0.018 | -0.195 | 0.176 | -0.001 | 0.096-0.254 | = | 0.195 | -0.011 | 0.137-0.252 | = |
