# Supplementary material for: Forelimb musculoskeletal-tendinous growth in frogs
Source: PeerJ. 2020 Feb 25;8:e8618. doi: 10.7717/peerj.8618 (PMC7047859; doi:10.7717/peerj.8618)
Supplement: Table S5 [file peerj-08-8618-s005.docx]

| Variables | Expected allometry coefficient | Observed allometry coefficient | Observed departure | Untrimmed | | | | Trimmed | | | |
| --- | --- | --- | --- | --- | --- | --- | --- | --- | --- | --- | --- |
|  |  |  |  | Resampled allometry coefficient | Bias | 95 % CI | Growth trend | Resampled allometry coefficient | Bias | 95 % CI | Growth trend |
| LT | 0.21 | 0.864 | 0.651 | 0.226 | -0.003 | 0.208-0.244 | = | 0.229 | -0.004 | 0.219-0.239 | + |
| HL | 0.21 | 0.217 | 0.004 | 0.274 | -0.004 | 0.234-0.314 | + | 0.248 | 0.009 | 0.233-0.264 | + |
| RUL | 0.21 | 0.166 | -0.047 | 0.284 | -0.005 | 0.238-0.329 | + | 0.279 | -0.002 | 0.245-0.313 | + |
| SM | 0.21 | 0.137 | -0.076 | 0.245 | -0.002 | 0.211-0.278 | = | 0.250 | -0.004 | 0.222-0.278 | + |
| SMTL | 0.21 | 0.060 | -0.153 | 0.163 | 0.002 | 0.083-0.242 | = | 0.208 | -0.020 | 0.175-0.241 | = |
| Hlat | 0.21 | 0.141 | -0.072 | 0.255 | -0.004 | 0.188-0.322 | = | 0.215 | 0.017 | 0.191-0.238 | = |
| HlatTL | 0.21 | 0.045 | -0.168 | 0.173 | 0.005 | 0.029-0.318 | = | 0.177 | 0.003 | 0.083-0.270 | = |
| Hmed | 0.21 | 0.073 | -0.140 | 0.185 | -0.003 | 0.091-0.280 | = | 0.165 | 0.007 | 0.120-0.209 | - |
| HmedTL | 0.21 | 0.018 | -0.195 | 0.124 | 0.000 | 0.046-0.201 | - | 0.110 | 0.007 | 0.067-0.152 | - |
| Edig | 0.21 | 0.162 | -0.051 | 0.262 | 0.001 | 0.211-0.312 | = | 0.253 | 0.005 | 0.221-0.285 | + |
| Ecul | 0.21 | 0.114 | -0.099 | 0.230 | -0.002 | 0.163-0.296 | = | 0.226 | 0.000 | 0.192-0.260 | = |
| EculT | 0.21 | 0.018 | -0.195 | 0.110 | -0.001 | 0.048-0.171 | - | 0.069 | 0.019 | 0.046-0.093 | - |
| Ecr | 0.21 | 0.085 | -0.129 | 0.179 | -0.001 | 0.137-0.220 | = | 0.168 | 0.004 | 0.137-0.199 | - |
| EcrT | 0.21 | 0.015 | -0.198 | 0.090 | -0.002 | 0.032-0.147 | - | 0.083 | 0.002 | 0.039-0.126 | - |
| C | 0.21 | 0.126 | -0.087 | 0.250 | -0.004 | 0.207-0.292 | = | 0.233 | 0.005 | 0.218-0.247 | + |
| CTL | 0.21 | 0.178 | -0.035 | 0.285 | -0.003 | 0.262-0.308 | + | 0.278 | 0.000 | 0.261-0.294 | + |
| Fdc | 0.21 | 0.107 | -0.106 | 0.202 | 0.000 | 0.161-0.242 | = | 0.201 | 0.000 | 0.175-0.226 | = |
| FdcT | 0.21 | 0.051 | -0.162 | 0.215 | 0.001 | 0.159-0.270 | = | 0.219 | -0.001 | 0.177-0.259 | = |
| Fcul | 0.21 | 0.080 | -0.133 | 0.200 | -0.003 | 0.159-0.241 | = | 0.190 | 0.002 | 0.1600.220 | = |
| FculT | 0.21 | 0.018 | -0.195 | 0.164 | -0.005 | 0.089-0.239 | = | 0.171 | -0.008 | 0.104-0.238 | = |
| Fcr | 0.21 | 0.109 | -0.104 | 0.240 | -0.002 | 0.204-0.276 | = | 0.227 | 0.004 | 0.206-0.248 | = |
| FcrT | 0.21 | 0.028 | -0.185 | 0.260 | -0.003 | 0.208-0.313 | = | 0.245 | 0.005 | 0.216-0.274 | + |
