# Supplementary material for: Forelimb musculoskeletal-tendinous growth in frogs
Source: PeerJ. 2020 Feb 25;8:e8618. doi: 10.7717/peerj.8618 (PMC7047859; doi:10.7717/peerj.8618)
Supplement: Table S6 [file peerj-08-8618-s006.docx]

| Variable | Expected allometry coefficient | Observed  allometry coefficient | | Observed departured | | Untrimmed | | |  | | |  | |  |  | | Trimmed | | | | |  |  |  |  |  |  |  |  |  |  |  |  |  |  |  |  |  |  |  |  |
| --- | --- | --- | --- | --- | --- | --- | --- | --- | --- | --- | --- | --- | --- | --- | --- | --- | --- | --- | --- | --- | --- | --- | --- | --- | --- | --- | --- | --- | --- | --- | --- | --- | --- | --- | --- | --- | --- | --- | --- | --- | --- |
|  |  |  | |  | | Resampled allometry coefficient | Bias | | 95 % CI | | | Growth  trend | | |  | | Resampled allometry coefficient | | Bias | | | | | 95 % CI | | Growth trend | | |  |  |  |  |  |  |  |  |  |  |  |  |  |
| LT | 0.21 | 0.795 | | 0.582 | | 0.227 | -0.003 | | 0.215-0.238 | | | + | | |  | | 0.226 | | -0.002 | | | | | 0.219-0.234 | | + | | |  | | |  |  |  |  |  |  |  |  |  |  |
| HL | 0.21 | 0.258 | | 0.045 | | 0.185 | -0.002 | | 0.144-0.225 | | | = | | |  | | 0.182 | | -0.001 | | | | | 0.148-0.217 | | = | | |  | | |  |  |  |  |  |  |  |  |  |  |
| RUL | 0.21 | 0.149 | | -0.064 | | 0.236 | -0.002 | | 0.211-0.260 | | | + | | |  | | 0.237 | | -0.003 | | | | | 0.222-0.252 | | = | | |  | | |  |  |  |  |  |  |  |  |  |  |
| SM | 0.21 | 0.220 | | 0.007 | | 0.248 | -0.002 | | 0.212-0.284 | | | + | | |  | | 0.251 | | -0.003 | | | | | 0.223-0.278 | | = | | |  | | |  |  |  |  |  |  |  |  |  |  |
| SMTL | 0.21 | 0.027 | | -0.186 | | 0.283 | -0.005 | | 0.180-0.385 | | | + | | |  | | 0.291 | | -0.009 | | | | | 0.220-0.362 | | = | | |  | | |  |  |  |  |  |  |  |  |  |  |
| Hlat | 0.21 | 0.183 | | -0.030 | | 0.254 | -0.003 | | 0.236-0.272 | | | + | | |  | | 0.251 | | -0.001 | | | | | 0.237-0.264 | | + | | |  | | |  |  |  |  |  |  |  |  |  |  |
| HlatTL | 0.21 | 0.021 | | -0.192 | | 0.116 | -0.001 | | 0.010-0.222 | | | - | | |  | | 0.134 | | -0.010 | | | | | 0.059-0.209 | | = | | |  | | |  |  |  |  |  |  |  |  |  |  |
| Hmed | 0.21 | 0.134 | | -0.079 | | 0.274 | -0.003 | | 0.215-0.333 | | | + | | |  | | 0.261 | | 0.004 | | | | | 0.214-0.307 | | + | | |  | | |  |  |  |  |  |  |  |  |  |  |
| HmedTL | 0.21 | 0.030 | | -0.183 | | 0.178 | -0.007 | | 0.025-0.331 | | | - | | |  | | 0.092 | | 0.036 | | | | | 0.008-0.177 | | = | | |  | | |  |  |  |  |  |  |  |  |  |  |
| Edig | 0.21 | 0.153 | | -0.060 | | 0.255 | -0.003 | | 0.235-0.275 | | | + | | |  | | 0.254 | | -0.002 | | | | | 0.237-0.270 | | + | | |  | | |  |  |  |  |  |  |  |  |  |  |
| Ecul | 0.21 | 0.134 | | -0.079 | | 0.290 | -0.003 | | 0.259-0.322 | | | + | | |  | | 0.295 | | -0.006 | | | | | 0.274-0.317 | | + | | |  | | |  |  |  |  |  |  |  |  |  |  |
| EculT | 0.21 | 0.007 | | -0.206 | | 0.198 | -0.003 | | 0.115-0.281 | |  | | = | | | 0.198 | |  | |  | -0.003 0.144-0.252 = | | | |  | | |  | |  |  |  |  |  |  |  |  |  |  |  |  |
| Ecr | 0.21 | 0.077 | | -0.137 | | 0.209 | -0.001 | | 0.139-0.279 | | | = | | |  | | 0.217 | | -0.005 | | | | | 0.169-02.64 | | = | | |  | | |  |  |  |  |  |  |  |  |  |  |
| EcrT | 0.21 | 0.015 | | -0.198 | | 0.276 | -0.004 | | 0.189-0.363 | | | + | | |  | | 0.315 | | -0.023 | | | | | 0.251-0.379 | | = | | |  | | |  |  |  |  |  |  |  |  |  |  |
| C | 0.21 | 0.179 | | -0.034 | | 0.185 | -0.002 | | 0.149-0.221 | | | - | | |  | | 0.174 | | 0.003 | | | | | 0.148-0.200 | | = | | |  | | |  |  |  |  |  |  |  |  |  |  |
| CTL | 0.21 | 0.153 | | -0.060 | | 0.212 | -0.002 | | 0.190-0.234 | | | = | | |  | | 0.217 | | -0.004 | | | | | 0.200-0.233 | | = | | |  | | |  |  |  |  |  |  |  |  |  |  |
| Fdc | 0.21 | 0.138 | | -0.075 | | 0.269 | -0.002 | | 0.246-0.291 | | | + | | |  | | 0.272 | | -0.004 | | | | | 0.253-0.291 | | + | | |  | | |  |  |  |  |  |  |  |  |  |  |
| FdcT | 0.21 | 0.030 | | -0.184 | | 0.028 | -0.001 | | -0.072-0.128 | | | - | | |  | | 0.021 | | 0.003 | | | | | -0.059-0.101 | | - | | |  | | |  |  |  |  |  |  |  |  |  |  |
| Fcul | 0.21 | 0.105 | | -0.108 | | 0.238 | -0.002 | | 0.197-0.278 | | | + | | |  | | 0.245 | | -0.006 | | | | | 0.220-0.270 | | = | | |  | | |  |  |  |  |  |  |  |  |  |  |
| FculT | 0.21 | 0.008 | | -0.206 | | -0.068 | 0.044 | | -0.166-0.030 | | | - | | |  | | -0.068 | | 0.044 | | | | | -0.144-0.008 | | - | | |  | | |  |  |  |  |  |  |  |  |  |  |
| Fcr | 0.21 | 0.199 | | -0.014 | | 0.245 | -0.003 | | 0.218-0.271 | | | + | | |  | | 0.235 | | 0.002 | | | | | 0.219-0.251 | | + | | |  | | |  |  |  |  |  |  |  |  |  |  |
| FcrT | 0.21 | 0.052 | | -0.161 | | 0.071 | 0.000 | | 0.003-0.139 | | | - | | |  | | 0.061 | | 0.004 | | | | | 0.009-0.114 | | - | | |  | | |  |  |  |  |  |  |  |  |  |  |
|  | | |  | |  | | |  | |  | | | | | | | | |  | | | | | | | |  | | | |  | |  |  |  | -0.003405399 | 0.25203492 | 0.14436758 | ISO | ISO | EculT |
