# Supplementary material for: Forelimb musculoskeletal-tendinous growth in frogs
Source: PeerJ. 2020 Feb 25;8:e8618. doi: 10.7717/peerj.8618 (PMC7047859; doi:10.7717/peerj.8618)
Supplement: Table S7 [file peerj-08-8618-s007.docx]

| Variables | Expected allometry coefficient | Observed allometry coefficient | Observed departure | Untrimmed | | | | Trimmed | | | |
| --- | --- | --- | --- | --- | --- | --- | --- | --- | --- | --- | --- |
|  |  |  |  | Resampled allometry coefficient | Bias | 95 % CI | Growth trend | Resampled allometry coefficient | Bias | 95 % CI | Growth trend |
| LT | 0.21 | 0.835 | 0.622 | 0.228 | -0.002 | 0.194-0.262 | = | 0.229 | -0.004 | 0.219-0.239 | + |
| HL | 0.21 | 0.196 | -0.017 | 0.205 | -0.003 | 0.176-0.233 | = | 0.248 | 0.009 | 0.233-0.264 | + |
| RUL | 0.21 | 0.179 | -0.035 | 0.251 | -0.003 | 0.218-0.284 | + | 0.279 | -0.002 | 0.245-0.313 | + |
| SM | 0.21 | 0.141 | -0.073 | 0.221 | -0.001 | 0.168-0.272 | = | 0.250 | -0.004 | 0.222-0.278 | + |
| SMTL | 0.21 | 0.048 | -0.165 | 0.156 | -0.005 | 0.067-0.244 | = | 0.208 | -0.020 | 0.175-0.241 | = |
| Hlat | 0.21 | 0.169 | -0.044 | 0.267 | -0.002 | 0.220-0.314 | + | 0.215 | 0.017 | 0.191-0.238 | = |
| HlatTL | 0.21 | 0.031 | -0.182 | 0.157 | -0.003 | 0.077-0.237 | = | 0.177 | 0.003 | 0.083-0.270 | = |
| Hmed | 0.21 | 0.100 | -0.113 | 0.215 | -0.002 | 0.172-0.257 | = | 0.165 | 0.007 | 0.120-0.209 | - |
| HmedTL | 0.21 | 0.017 | -0.197 | 0.174 | 0.000 | 0.091-0.256 | = | 0.110 | 0.007 | 0.067-0.152 | - |
| Edig | 0.21 | 0.225 | 0.012 | 0.327 | -0.004 | 0.271-0.384 | + | 0.253 | 0.005 | 0.221-0.285 | + |
| Ecul | 0.21 | 0.153 | -0.060 | 0.251 | -0.003 | 0.219-0.283 | + | 0.226 | 0.000 | 0.192-0.260 | = |
| EculT | 0.21 | -0.003 | -0.216 | 0.041 | -0.002 | (-0.034)-0.116 | - | 0.069 | 0.019 | 0.046-0.093 | - |
| Ecr | 0.21 | 0.099 | -0.114 | 0.208 | -0.002 | 0.157-0.258 | = | 0.168 | 0.004 | 0.137-0.199 | - |
| EcrT | 0.21 | 0.043 | -0.170 | 0.286 | -0.005 | 0.161-0.411 | = | 0.083 | 0.002 | 0.039-0.126 | - |
| C | 0.21 | 0.095 | -0.118 | 0.177 | -0.003 | 0.127-0.227 | = | 0.233 | 0.005 | 0.218-0.247 | + |
| CTL | 0.21 | 0.160 | -0.053 | 0.226 | -0.003 | 0.195-0.257 | = | 0.278 | 0.000 | 0.261-0.294 | + |
| Fdc | 0.21 | 0.146 | -0.067 | 0.252 | -0.003 | 0.198-0.305 | = | 0.201 | 0.000 | 0.175-0.226 | = |
| FdcT | 0.21 | 0.019 | -0.194 | 0.124 | -0.001 | 0.032-0.215 | = | 0.219 | -0.001 | 0.177-0.259 | = |
| Fcul | 0.21 | 0.119 | -0.095 | 0.234 | -0.002 | 0.159-0.309 | = | 0.190 | 0.002 | 0.160-0.220 | = |
| FculT | 0.21 | 0.026 | -0.187 | 0.204 | 0.001 | 0.067-0.340 | = | 0.171 | -0.008 | 0.104-0.238 | = |
| Fcr | 0.21 | 0.113 | -0.100 | 0.205 | -0.002 | 0.166-0.243 | = | 0.227 | 0.004 | 0.206-0.248 | = |
| FcrT | 0.21 | 0.020 | -0.193 | 0.217 | -0.006 | 0.145-0.287 | = | 0.245 | 0.005 | 0.216-0.274 | + |
